# Activated gut-homing CD8^+^ T cells for coeliac disease diagnosis on a gluten-free diet

**DOI:** 10.1186/s12916-021-02116-z

**Published:** 2021-10-06

**Authors:** Fernando Fernández-Bañares, Natalia López-Palacios, María Corzo, Beatriz Arau, Mercedes Rubio, Marta Fernández-Prieto, Eva Tristán, Mar Pujals, Sergio Farrais, Saúl Horta, Juana María Hernández, Marta Gomez-Perosanz, Pedro A. Reche, María Esteve, Concepción Núñez

**Affiliations:** 1 Department of Gastroenterology, Hospital Universitari Mutua Terrassa, Terrassa, Barcelona Spain; 2 Centro de Investigación Biomédica en Red de Enfermedades Hepáticas y Digestivas (CIBERehd), Instituto de Salud Carlos III, Madrid, Spain; 3 Servicio de Aparato Digestivo, Hospital Clínico San Carlos, Instituto de Investigación Sanitaria del Hospital Clínico San Carlos (IdISSC), 28040 Madrid, Spain; 4 Laboratorio de Investigación en Genética de enfermedades complejas, Hospital Clínico San Carlos, Instituto de Investigación Sanitaria del Hospital Clínico San Carlos (IdISSC), 28040 Madrid, Spain; 5 Servicio de Aparato Digestivo, Hospital Universitario Fundación Jiménez Díaz, 28040 Madrid, Spain; 6 Facultad de Medicina, Laboratorio de Inmunomedicina, Departamento de Inmunología, Universidad Complutense de Madrid, 28040 Madrid, Spain

**Keywords:** CD8 T cells, Celiac disease, Gluten challenge, Gluten-free diet, IFN-γ ELISPOT, Flow cytometry

## Abstract

**Background:**

The diagnosis of coeliac disease (CD) in individuals that have started a gluten-free diet (GFD) without an adequate previous diagnostic work-out is a challenge. Several immunological assays such as IFN-γ ELISPOT have been developed to avoid the need of prolonged gluten challenge to induce the intestinal damage. We aimed to evaluate the diagnostic accuracy of activated gut-homing CD8^+^ and TCRγδ^+^ T cells in blood after a 3-day gluten challenge and to compare it with the performance of IFN-γ ELISPOT in a HLA-DQ2.5 subsample.

**Methods:**

A total of 22 CD patients and 48 non-CD subjects, all of them following a GFD, underwent a 3-day 10-g gluten challenge. The percentage of two T cell subsets (CD8^+^ CD103^+^ β7^hi^ CD38^+^/total CD8^+^ and TCRγδ^+^ CD103^+^ β7^hi^ CD38^+^/total TCRγδ^+^) in fresh peripheral blood drawn baseline and 6 days after the challenge was determined by flow cytometry. IFN-γ ELISPOT assays were also performed in HLA-DQ2.5 participants. ROC curve analysis was used to assess the diagnostic performance of the CD8^+^ T cell response and IFN-γ ELISPOT.

**Results:**

Significant differences between the percentage of the two studied subsets of CD8^+^ and TCRγδ^+^ cells at days 0 and 6 were found only when considering CD patients (*p* < 10^−3^ vs. non-CD subjects). Measuring activated CD8^+^ T cells provided accurate CD diagnosis with 95% specificity and 97% sensitivity, offering similar results than IFN-γ ELISPOT.

**Conclusions:**

The results provide a highly accurate blood test for CD diagnosis in patients on a GFD of easy implementation in daily clinical practice.

**Supplementary Information:**

The online version contains supplementary material available at 10.1186/s12916-021-02116-z.

## Background

Coeliac disease (CD) is a chronic immune-mediated systemic disease, which is triggered by gluten ingestion in genetically susceptible individuals. Clinical, serological, and histopathological data are used for CD diagnosis. However, these parameters generally normalized in patients following a gluten-free diet (GFD). In recent years, many patients come to the visit having started a GFD before a definitive CD diagnosis is established. In these patients, the diagnosis of CD can be problematic since a prolonged gluten challenge in a symptom-free patient on a GFD is always difficult. In recent years, low-invasive novel procedures have been proposed as good approaches for CD diagnosis in individuals following a GFD [1–9]. This would represent an important step in clinical practice, since it would make it easier to diagnose individuals with self-prescribed GFD and those needing review of the initial diagnosis due to incomplete original testing, discrepant results, or slow or non-responsiveness to the GFD.

Determination of IL-2 has been described as the earliest potential marker for CD diagnosis after a short gluten challenge [7]. Even a whole blood IL-2 and IFN-γ release assay has been very recently described for diagnosis with no challenge requirement [8, 9]. Additionally, a 3-day gluten challenge mobilizes gut-homing memory T cells that can be detected in peripheral blood using different technologies [1, 3, 5, 7]. IFN-γ ELISPOT assays and HLA-DQ2-gliadin tetramer staining have been the most extensively described approaches, showing high sensitivity and specificity. Cytometry by time-of-flight mass spectrometry (CyTOF) enables to assay numerous parameters (>40) simultaneously and provides a deep phenotypic characterization of the mobilized cells. However, all of them show cost and technological limitations for translation into clinical practice and their sensitivity decreases in non-HLA-DQ2.5 subjects [7, 10]. Flow cytometry constitutes a more realistic alternative. This technique has become a routine tool in clinical laboratories, with increasing use in disease diagnosis [11]. We previously demonstrated that CD patients could be diagnosed in individuals on a GFD after a 3-day gluten challenge by analyzing only four markers in peripheral blood by flow cytometry [3]. The selected markers allow us to identify activated (CD38) gut-homing (CD103 and β7) CD8 T cells. In the current study, we aimed to further explore this diagnostic approach analyzing additional patients and controls and giving measures of diagnostic accuracy. As a secondary aim, we performed a pilot study to compare this test with the production of IFN-γ measured by ELISPOT in a subgroup of HLA-DQ2.5 patients.

## Methods

### Subjects

A total of 70 individuals were included: 22 CD patients and 48 non-CD controls (Table 1).

**Table 1:** Characteristics of the participants in the study

	CD patients			Non-CD controls			
	Total	Marsh 3	Marsh 1	Total	Healthy	NR-GFD	R-GFD
	***N*** = 22	***N*** = 14	***N*** = 8	***N*** = 48	***N*** = 13	***N*** = 21	***N*** = 14
**Age**† **(years)**							
Mean ± SE	50.4 ± 3.5	49.3 ± 4.2	52.0 ± 6.4	38.3 ± 1.9	40.0 ± 3 .7	35.5 ± 2.5	40.5 ± 4.0
Range	24–83	24–69	31–83	16–63	23–63	16–49	19–62
**Age at diagnosis (years)**							
Mean ± SE	41.8 ± 3.8	40.1 ± 4.5	44.7± 7.1	--	--	--	--
Range	15–80	15–65	22–80				
**Females (*N*, %)**	16 (72.7)	10 (71)	6 (75)	37 (77.1)	10 (77)	14 (67)	13 (93)
**ATG2 positive (*N*, %)**	22 (100)‡	14 (100)‡	8 (100)	0	0	0	0
**HLA (*N*, %)**							
DQ2.5	20 (91.0)	13 (93)	7 (87.5)	21 (44.7)	8 (62)	10 (50)	3 (21)
DQ8	0	0	0	5 (10.6)	0	3 (15)	2 (14)
DQ2.2	1 (4.5)	1 (7)	0	7 (14.9)	1 (8)	2 (10)	4 (29)
DQ7.5	0	0	0	4 (8.5)	1 (8)	1 (5)	2 (14)
Non-DQ2/DQ8	1 (4.5)	0	1 (12.5)	10 (21.3)	3 (23)	4 (20)	3 (21)
**Histology (*N*, %)**§							
ND	0	0	0	16 (33.3)	13 (100)	0	3 (21)
Marsh 0	0	0	0	21 (43.8)	0	14 (67)	7 (50)
Marsh 1	8 (36.4)	0	8 (100)	11 (22.9)	0	6 (29)	4 (29)
Marsh 3a	5 (22.7)	5 (38)	0	0	0	0	0
Marsh 3b	4 (18.2)	4 (31)	0	0	0	0	0
Marsh 3c	4 (18.2)	4 (31)	0	0	0	0	0
**Time on GFD**							
Mean ± SE (years)	7.4 ± 1.3	8.0 ± 1.7	6.2 ± 2.0	1.8 ± 0.4	0.09 ± 0.005	2.6 ± 0.6	2.7 ± 0.8
Range	1–25 years	1–25 years	1–16 years	1 month–11 years	30–45 days	1 month–6 years	4 months–11 years

*Abbreviations*: *CD*, coeliac disease; *GFD*, gluten-free diet; *NR-GFD*, disease controls with no clinical response to a GFD; *R-GFD*, disease controls with a clinical response to a GFD; *ND*, not done; *ATG2*, antibodies against transglutaminase type 2; DQ2.5 = DQB1*02 and DQA1*05; DQ8 = DQB1*03:02-DQA1*03; DQ2.2 = DQB1*02:02-DQA1*02:01; DQ7.5 = DQB1*03:01-DQA1*05

†Age at the moment of the gluten challenge; ‡one patient only had anti-endomysial antibodies tested (with a positive result); §one patient had Marsh 3 with no information about the subtype

CD patients were adults with diagnosis based on ESPGHAN and UEG criteria [12, 13]. All showed positive serology (anti-transglutaminase type 2 (TG2) antibodies but one patient diagnosed 27 years ago with positive IgA anti-gliadin and anti-endomysial antibodies) and enteropathy (Marsh 3 or Marsh 1 lesion) at diagnosis (on a gluten-containing diet), with a clinical and serological response to a GFD. At the time of the study, they were asymptomatic and showed negative anti-TG2 serology. Of note, one patient lacked any HLA risk allele and showed HLA-DQA1*03:02-HLA-DQB1*03:03 (haplotype HLA-DQ9.3)/HLA-DQA1*01:02-HLA-DQB1*06:02. Anti-TG2 titers were obtained using a quantitative automated ELISA (Elia CelikeyTM, Phadia AB, Freiburg, Germany). The manufacturer’s recommended cut-off for anti-TG2 was 8 U/mL, but in order to increase the sensitivity of the serological assay, it was reduced to 2 (98% of individuals showed values below 2 U/mL in a population-based study) [14].

The control groups comprised adult individuals on a GFD. CD was discarded before GFD introduction by means of negative anti-TG2 serology accompanied, in those with clinical symptoms, of normal histology and/or negative HLA genetics. Specifically, non-CD groups included the following:

1. Healthy subjects (*N* = 13): CD relatives or staff members of the participating hospitals with no clinical symptoms who voluntarily followed a GFD for at least the prior month. All showed negative coeliac serology just before starting the GFD.

2. Disease subjects with lack of clinical response to a GFD (NR-GFD, *N* = 25): patients attending CD monographic outpatients visit mainly by functional dyspepsia or symptoms compatible with irritable bowel syndrome who followed a GFD by their own choice or by erroneous diagnosis. All had negative coeliac serology and showed normal histology except nine with minor histological alterations (Marsh 1) when following a gluten-containing diet. All these Marsh 1 patients had an absence of the intraepithelial celiac lymphogram [15, 16].

3. Disease subjects with a clinical response to a GFD (R-GFD, *N* = 14): subjects with suspicion of gluten-related functional bowel disease symptoms, negative coeliac serology, and non-compatible findings in the duodenal biopsy (7 patients showed Marsh 0 and 4 patients showed Marsh 1 with the absence of the intraepithelial celiac lymphogram [15, 16]) or non-CD predisposing HLA genetics (negative HLA-DQ2.5/DQ8/DQ2.2) (3 patients).

Participants using immunomodulatory medication or reporting previous severe acute reaction to involuntary gluten ingestion were excluded.

The study protocol was approved by the Ethical Committees of the participating hospitals (C.I. 17/181-E and Acta 02/17). Written informed consent was obtained from all the studied subjects.

### Gluten challenge

All participants followed a strict GFD for at least 30 days; then, they underwent a 3-day gluten challenge consisting of 160 g of gluten-containing sliced white bread (approximately 10–12 g of gluten) daily for three consecutive days (days 1–3). All participants completed the 3-day challenge.

The correct adherence to the GFD prior to the gluten challenge was determined by assessing the excretion of gluten immunogenic peptides (GIP) in stool or urine in all subject groups, except for the NR-GFD disease controls who could not be tested. In case of a positive GIP test (two healthy controls), additional 15 days on a very strict GFD and a negative GIP test were established prior to the study.

### Clinical response to the gluten challenge

Patients were asked to rate the most common clinical symptoms presented during the 6 days after gluten challenge (flatulence, abdominal distension, abdominal pain, altered bowel habits, asthenia, irritability, vomiting) according to a visual analogue scale (VAS) ranging from no symptoms (0) to very important (100) [17]. The presence/absence of clinical symptoms on day 6 after starting the gluten challenge was also recorded.

### Flow cytometry studies in blood

Peripheral blood was collected in EDTA tubes before starting gluten intake (day 0) and after 6 days (day 6) and processed in fresh (350 μl per subject per day) as previously described with minor modifications [3] (Additional file 1: Materials S1). After processed all the samples, data analysis was done blinded to the diagnostic result with the Kaluza Analysis Software (Beckman Coulter, CA, USA) using Batch Processing (Additional file 2: Figure S1).

### IFN-γ ELISPOT (enzyme-linked immunospot) assay

IFN-γ ELISPOT assays were performed as previously described [3] (Additional file 3: Materials S2) [18], except that the assayed PBMCs were isolated by Ficoll/Hypaque (StemCell Technologies) density-gradient centrifugation or by using Cell Preparation Tubes (CPT). Only patients carrying HLA-DQ2.5 were included for this analysis, which was performed in a subsample of 10 CD patients and 11 non-CD subjects.

### Additional methods

Histological and flow cytometry studies of the duodenal mucosa performed for the diagnosis are described in Additional file 3: Materials S3 [15, 16, 19, 20].

### Statistical analysis

Results are expressed as median (IQR) or mean (SE), and as percentages with their 95% CI when appropriate.

The percentages of the selected T cell populations in blood (CD8^+^ CD103^+^ β7^hi^ CD38^+^ and TCRγδ^+^ CD103^+^ β7^hi^ CD38^+^ T cells, regarding the total number of CD8^+^ and TCRγδ^+^ cells, respectively) were obtained for all the participants at day 0 (baseline) and at day 6. Comparisons within CD and non-CD groups were performed by using the one-sided Wilcoxon signed-rank test. Comparisons between both groups to evaluate the percentage of the selected T cell populations at day 6 and the ratio of the percentage at day 6/day 0 were performed by using the Mann-Whitney *U* test.

To analyze the IFN-γ ELISPOT data, the mean response of the triplicate negative control (medium) wells was subtracted from the triplicate p57-73 QE65 peptide wells in all samples. After that, the response to the gliadin peptide was calculated based on the difference between SFC/10^6^ cells obtained at day 6 and baseline by using the one-sided Wilcoxon signed-rank test.

The effectiveness for CD diagnosis of the T cell response measured by flow cytometry and IFN-γ ELISPOT was assessed by receiver operating characteristic (ROC) curve analysis using pROC packages in R version 4.0.3. Comparison of the area under the curve (AUC) of the ROC curves was performed using De Long’s test part of the pROC package. Sensitivity, specificity, and the positive and negative likelihood ratio with their 95% CI were also obtained by using the MedCalc statistical software. A CD prevalence of 1% was considered for calculations. For comparative purposes, these calculations were performed in the subsample of HLA-DQ2.5 patients with data for both CD8^+^ T cell response and IFN-γ ELISPOT. Additionally, in order to increase the accuracy of the estimated parameters, CD8^+^ T cell-based calculations were also obtained considering all the participants in this study together with those included in our previous publication [3].

Spearman’s rank correlation was calculated to know the relationship between pairs of quantitative variables.

The influence of the clinical response to the gluten challenge on the CD8^+^ T cell response was assessed by analyzing the correlation between the intensity of clinical symptoms and the percentage at day 6 of the CD8^+^ CD103^+^ β7^hi^ CD38^+^ regarding total CD8^+^ and by comparing the median of that percentage in the two groups established according to the presence/absence of clinical symptoms at day 6.

Graphs were performed with R version 4.0.3 using the ggplot2 and pROC packages.

## Results

### T cell response by flow cytometry

**Fig. 1:**
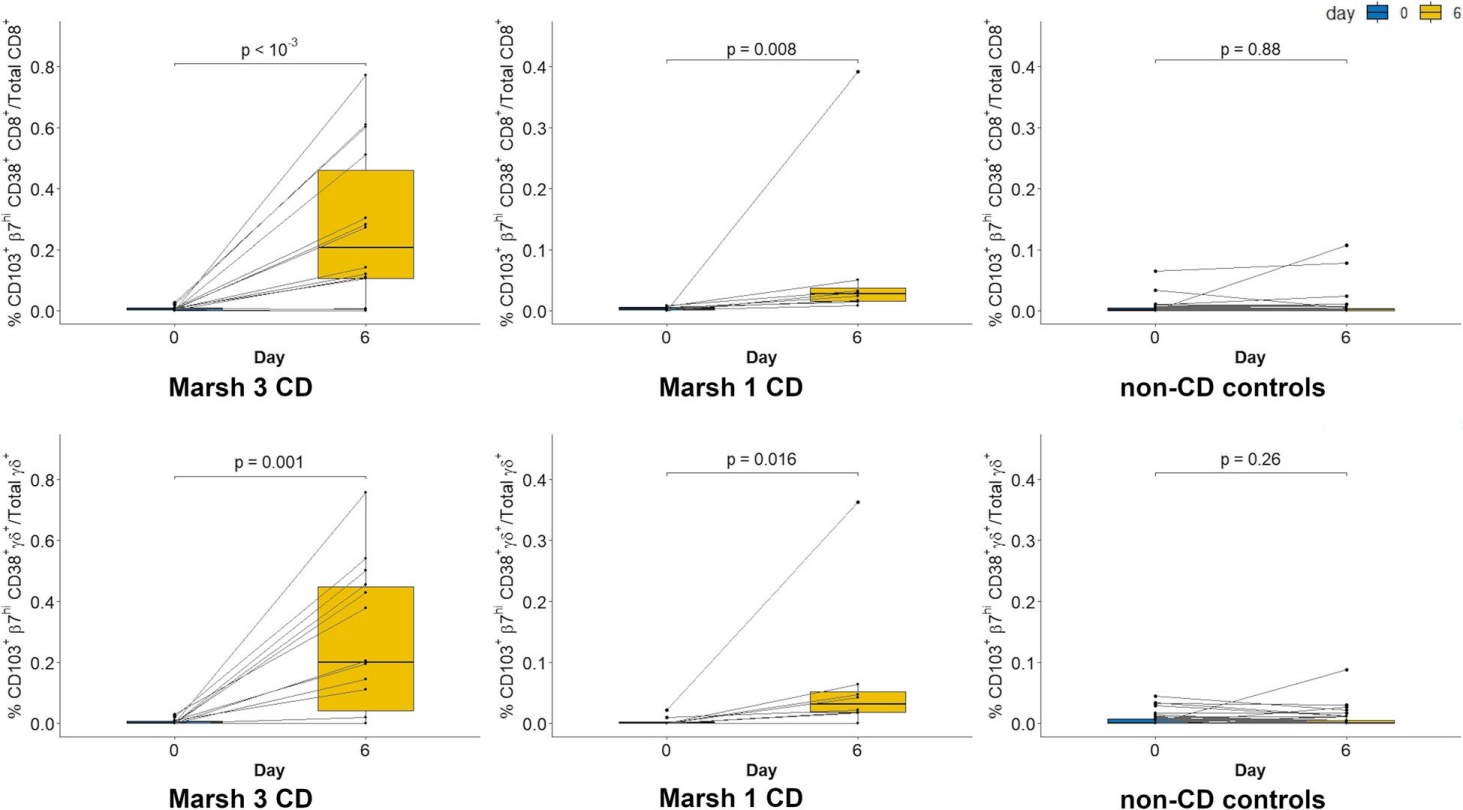
CD8^+^ (graphs above) and TCRγδ^+^ (graphs below) T cell response in peripheral blood after the 3-day gluten challenge in coeliac disease (CD) patients and non-CD controls. CD patients were subdivided depending on the histological characteristics (Marsh 1/Marsh 3). Lines connect results from individual patients. Centerlines in the box show the median; box limits indicate the 25th and 75th percentiles (IQR); whiskers extend to the highest and lowest observation excluding outliers

Significant differences between the percentage of the two studied cell types (CD8^+^ CD103^+^ β7^hi^ CD38^+^ and TCRγδ^+^ CD103^+^ β7^hi^ CD38^+^ T cells, regarding the total number of CD8^+^ and TCRγδ^+^ cells) at days 0 and 6 were found only when considering CD patients (Fig. 1).

As can be observed in Fig. 1, quite similar percentages of the studied CD8^+^ and TCRγδ^+^ T cell subpopulations appear at day 6 in CD patients: Spearman rho values = 0.72, *p* < 10^−3^. However, great differences exist when considering their absolute cell numbers. The average number of CD8^+^ T cells in the studied blood samples was around 77,400. However, when considering TCRγδ^+^ cells, the average number was approximately 10,900, being as low as 1565 in some participants. Consequently, the observed percentages of the studied TCRγδ^+^ T cells at day 6 were obtained with values of 5 cells or even less in the numerator (TCRγδ^+^ CD103^+^ β7^hi^ CD38^+^ T cells/total TCRγδ^+^ T cells) in some CD patients. Up to 17% of non-CD individuals showed between 2 and 5 TCRγδ^+^ CD103^+^ β7^hi^ CD38^+^ T cells at day 0; therefore, no conclusions should be drawn from very low cell numbers after gluten challenge for TCRγδ^+^ T cells.

At baseline, the selected T cell populations were absent or present in very low numbers in all participants: range 0–0.2, median 0.006 ± 0.001 for CD8^+^ CD103^+^ β7^hi^ CD38^+^/total CD8^+^ T cells and range 0–0.03, median 0.005 ± 0.002 for TCRγδ^+^ CD103^+^ β7^hi^ CD38^+^/ total TCRγδ^+^ T cells. Only six individuals showed a basal percentage of the selected CD8^+^ T cell population higher than 0.01%: three CD patients and three non-CD controls (all from the NR-GFD group). Notoriously, these percentages only increased after gluten challenge in CD patients. Therefore, the T cell response observed in CD is characterized by both: visualization of the studied T cell population after gluten challenge above a threshold (see calculations below) and a ratio day 6/day 0 ≥ 2. Comparison of these parameters between CD patients and non-CD controls showed significant differences (Table 2 and Additional file 3: Table S1).

**Table 2:** Median (IQR) of the studied CD8^+^ (percentage at day 6 and ratio day 6/day 0) and TCRγδ^+^ (percentage at day 6) T cell populations in CD patients and non-CD controls. Comparisons were performed with the Mann-Whitney *U* test

	% CD8^**+**^ CD103^**+**^ β7^**hi**^ CD38^**+**^/total CD8^**+**^	Ratio day 6/day 0	% TCRγδ^**+**^ CD103^**+**^ β7^**hi**^ CD38^**+**^/total TCRγδ^**+**^
**CD patients**	0.107 (0.018–0.297)	14.18 (6.68–33.55)	0.087 (0.018–0.373)
**Controls**	0.001 (0.000–0.003)	0.608 (0.000–1.191)	0.000 (0.000–0.005)
***p*-value**	1.11 × 10^−10^	1.47 × 10^−7^	7.26 × 10^−8^

It is noteworthy that an increased percentage of CD8^+^ CD103^+^ β7^hi^ CD38^+^ T cells after gluten challenge was observed in the patient lacking any HLA risk allele: from 0.003% at day 0 to 0.024% at day 6. Only one CD patient diagnosed with Marsh 3 showed zero CD8^+^ CD103^+^ β7^hi^ CD38^+^ T cells at both days 0 and 6. He had been following a GFD for 25 years and, since this lapse of time could preclude a response to a 3-day gluten challenge, he was excluded from subsequent calculations of diagnostic accuracy (see the “Discussion” section).

**Fig. 2:**
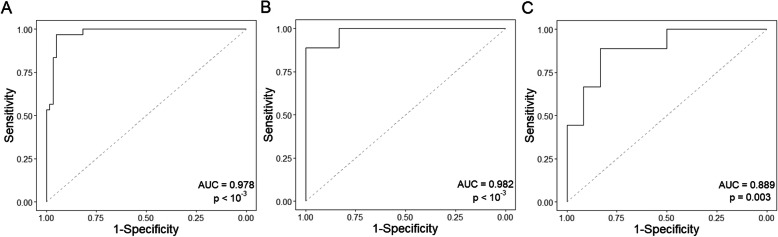
ROC curves for coeliac disease (CD) diagnosis based on the analysis of 30 CD vs. 60 non-CD subjects considering % CD8^+^ CD103^+^ β7^hi^ CD38^+^/total CD8^+^ T cells (**A**) and 9 CD vs. 12 non-CD subjects considering % CD8^+^ CD103^+^ β7^hi^ CD38^+^/total CD8^+^ T cells (**B**) or the difference in the number of IFN-γ SFC/10^6^ cells at day 6 and baseline by ELISPOT (**C**)

ROC curve analysis including these and our previous data [3] was performed to obtain the optimal cut-off point to diagnose CD based on the percentage of CD8^+^ CD103^+^ β7^hi^ CD38^+^/total CD8^+^ T cells (Fig. 2A, Table 3). Sensitivity was slightly lower than 100% (97%) due to one patient with a very low anti-TG2 level of 3.25 U/mL with positive anti-endomysial antibodies. The diagnostic values observed in Table 3 indicate a very good accuracy of the CD8^+^ T cell response to discriminate between CD and non-CD subjects.

**Table 3:** Diagnostic accuracy of the CD8 T^+^ cell response and IFN-γ ELISPOT

Parameter	Total	Subset assayed for both tests	
	CD8^**+**^ response	CD8^**+**^ response	IFN-γ ELISPOT
AUC (95% CI)	0.978 (0.953–1.003)†	0.982 (0.9345–1.028)	0.889 (0.747–1.031)
Optimal cut-off	0.006%	0.005%	28.35
Sensitivity	96.67 (82.78–99.92)	88.89 (51.75–99.72)	66.67 (29.93–92.51)
Specificity	95.0 (86.08–98.96)	100.00 (73.54–100.00)	91.67 (61.52–99.79)
Positive LR	19.33 (6.40–58.37)	–	8.0 (1.16–55.26)
Negative LR	0.04 (0.01–0.24)	0.11 (0.02–0.71)	0.36 (0.14–0.93)

*Abbreviations*: *AUC*, area under the curve; *LR*, likelihood ratio considering 1% of coeliac disease prevalence

According to the percentage of gut-homing CD8^+^ T cells observed after gluten challenge, three individuals out of 83 (3.6%) would be erroneously classified as CD patients, one from each control subgroup. The healthy control underwent duodenal biopsy in order to discard CD.

No correlation was found between the duration of the GFD prior to the inclusion in the study and the magnitude of the percentage of the studied CD8^+^ T cells.

### IFN-γ ELISPOT

**Fig. 3:**
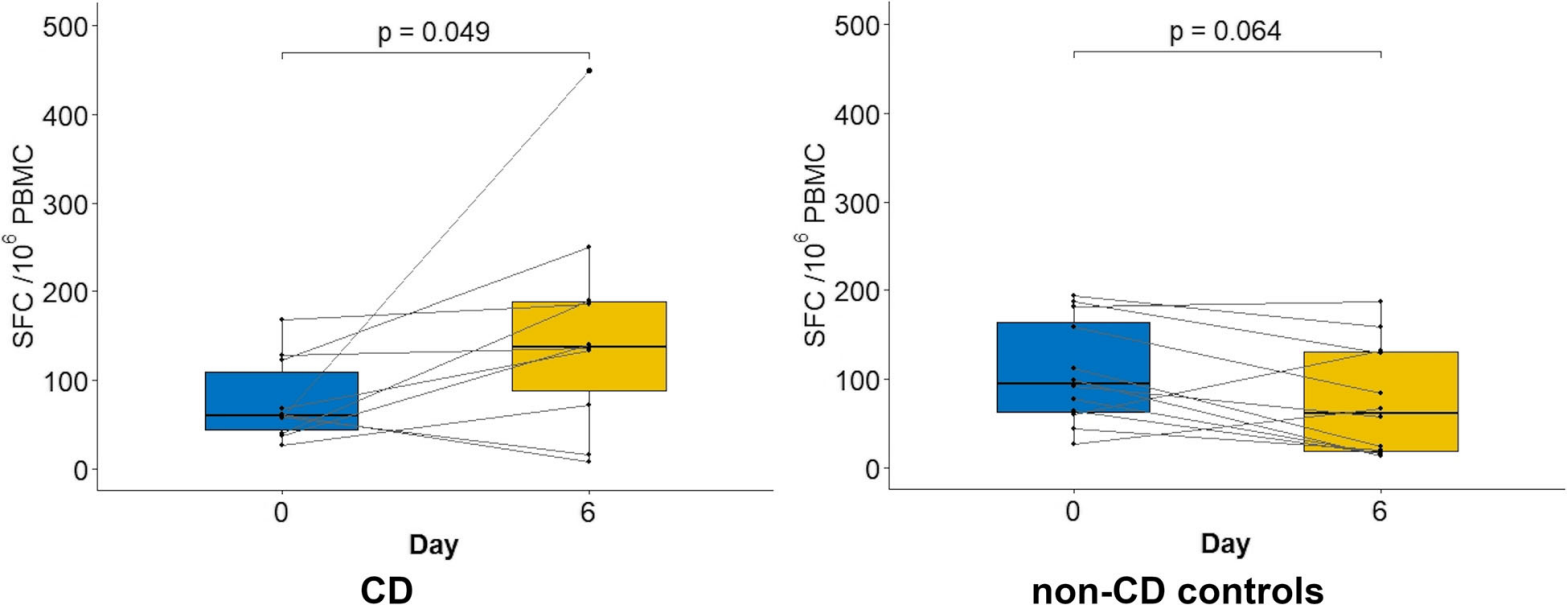
IFN-γ ELISPOT responses (spot forming cells (SFC)/10^6^ PBMC) to the 3-day gluten challenge measured baseline and at day 6 in the different groups of participants. CD, coeliac disease; NR-GFD, disease controls with no clinical response to a GFD; R-GFD, disease controls with a clinical response to a GFD. Lines connect results from individual patients. Centerlines in the box show the median; box limits indicate the 25th and 75th percentiles (IQR); whiskers extend to the highest and lowest observation excluding outliers

IFN-γ ELISPOT results are shown in Fig. 3. The patient following a GFD for 25 years also showed a negative result and was excluded from subsequent analyses. The ROC curve analysis considering CD patients vs. non-CD controls showed a slightly lower, although non-significant (*p* = 0.24), AUC than the obtained for the CD8^+^ T cell response in the same subset of patients (Fig. 2B, C). The optimal cut-off was observed for a difference between SFC/10^6^ cells obtained at day 6 and baseline ≥42.5 (86% sensitivity, 92% specificity). Three out of the 9 CD patients offered an IFN-γ ELISPOT response below that threshold, and 10 out the 11 non-CD controls, being these proportions similar to the obtained when testing the CD8^+^ T cell response. The patient with anti-TG2 = 3.25 U/mL at onset showed a positive response, although the lowest one considering either the value or the increase of SFC/10^6^ cells at day 6.

The difference between the IFN-γ SFC/10^6^ cells at day 6 and 0 did not correlate with the duration of the GFD prior to the gluten challenge.

### Clinical response

The clinical response to gluten challenge differed among the groups of participants (Additional file 3: Table S2, Additional file 4: Figure S2). Moderate-severe clinical symptoms were reported by a high proportion of R-GFD disease controls. Healthy controls were at the opposite end, but with very similar data to the ones reported by seropositive Marsh 1 CD patients. Around half of the healthy controls reported mild flatulence, abdominal distension, or altered bowel habits. Those three symptoms were also very frequently induced in NR-GFD disease controls, but appearing at higher severity and accompanied of abdominal discomfort. A high proportion of Marsh 3 seropositive patients reported gastrointestinal symptoms at a moderate mean severity. Vomiting was only reported by two seropositive patients. The two extra-digestive symptoms recorded (asthenia and irritability) were essentially reported by either CD or R-GFD disease control patients.

The magnitude of the CD8^+^ T cell response does not seem to be related to the reported clinical symptoms. No correlation was observed when considering the percentage of CD8^+^ CD103^+^ β7^hi^ CD38^+^/total CD8^+^ T cells and either any clinical symptom or the global VAS score. Only 38% of CD patients reported clinical symptoms 6 days after starting the gluten challenge. No significant differences were observed in the CD8^+^ T cell response when comparing CD patients showing or not clinical symptoms at day 6 (Mann-Whitney *U* test *p* = 0.92).

## Discussion

In this work, CD8^+^ and TCRγδ^+^ T cell responses were evaluated in blood samples from subjects who underwent a 3-day gluten challenge. Activated gut-homing CD8^+^ T cells (CD8^+^ CD103^+^ β7^hi^ CD38^+^) were observed in the peripheral blood of CD patients, providing a diagnostic tool reaching 95% of specificity and 97% of sensitivity. Even a sensitivity of 100% can be considered if the patient with a very low anti-TG2 titer (below the manufacturer’s recommended cut-off) is excluded. Therefore, similar values to the previously reported for IFN-γ ELISPOT, blood cytokine release assays, or HLA-DQ2-gliadin tetramers are obtained [2, 4], but avoiding the technical constraints.

This finding constitutes an important advance into clinical practice. Gluten challenge is proposed for CD diagnosis of individuals with self-prescribed GFD and it is cumbersome for most of them. Our study shows that a blood test based on the analysis of four markers (CD8, CD103, β7, and CD38) by flow cytometry in individuals exposed to a 3-day gluten challenge seems as accurate in patients on a GFD as it is anti-TG2 serology in patients on a normal diet.

Gluten challenge is also recommended in doubtful cases: individuals with a Marsh 1 lesion or subjects with low-risk or non-permissive HLA genetics. The test here proposed shows high accuracy to diagnose Marsh 1 CD, which could allow distinguishing this disease from false positive serological results associated with either an unspecific mucosal lesion or other pathologies [21, 22]. The HLA influence could not be extensively investigated due to the very low number of non-HLA-DQ2.5 studied CD patients. However, it is particularly striking the CD8^+^ T cell response to gluten challenge in one CD patient lacking any HLA risk allele. Previously, this cell response had been detected in one HLA-DQ2.2 and one HLA-DQ8 patient [3, 23]. This could represent a notable advantage of the CD8^+^ T cell-based blood test when compared to ELISPOT, HLA-DQ2-gliadin tetramers technologies, and the recent proposed cytokine release assays [1, 5, 9], which require the use of selected gliadin peptides. CD patients respond to distinct gluten peptides, which imposes limits when testing the immunological response to specific gluten peptides. HLA genetic risk lies on the capacity of gluten peptides to form kinetically stable complex with the encoded HLA-DQ receptors, which is needed to elicit the T cell immune response leading to CD. It is beyond the scope of this work to speculate how CD can take place with the non-permissive HLA genetics, but once produced, CD is probably developed following the characteristic immunological cascade. The haplotype HLA-DQA1*03-DQB1*03:03 (HLA-DQ9.3), present in the studied CD subject lacking HLA risk, can reach considerable frequency in China, where it has been described as a CD susceptibility factor [24]. HLA-DQ9.3-restricted gluten-reactive T cells were detected in the small intestine of a CD patient [25]. Now, we show that they can also be detected in the blood.

Our work also solves an outstanding issue in the clinical practice, the amount and duration of dietary gluten necessary to elicit a measurable response. A 3-day challenge with 10 g of gluten allows accurate diagnosis. Some works have suggested that lower amounts of gluten can elicit changes in CD patients, thereby reducing possible discomfort and risk. However, a lower percentage of patients showing gut-homing activated CD8^+^ T cells was recently observed in patients enrolled in a 3-g vs. 10-g 3-day gluten challenge (17% vs. 83%) [7], although PBMCs instead of whole blood were tested, which could decrease sensitivity [26, 27].

Our results warn clinicians to be aware that symptoms developed after gluten challenge are unreliable indicators of the presence of CD since flatulence, abdominal distention, or altered bowel habits were also frequently reported by both healthy and disease controls, which is concordant with the literature [28]. It must be noted that most of our R-GFD disease controls probably show non-coeliac gluten sensitivity, but the diagnosis of this condition is complex, and our patients did not undergo a double-blind, placebo-controlled gluten challenge as suggested to establish a firm diagnosis [29]. Nevertheless, one limitation of our work is the use of white bread for the challenge, which can make that non-gluten dietary components such as fermentable carbohydrates (FODMAPs) confound clinical symptoms.

The CD8^+^ T cell response did not correlate with the intensity of the clinical symptoms triggered by the gluten challenge. This may seem to contrast with the correlation reported between symptom severity and cytokine levels, mainly IL-2, elicited after gluten challenge [6, 10]. However, that correlation was particularly evident for nausea and vomiting, two symptoms present in only two CD patients of our sample. This could be again related to the non-specificity of symptoms caused by the use of bread for the challenge. In any case, it is noteworthy that the present diagnostic approach is valid for subjects with an asymptomatic response to the gluten challenge.

The lack of correlation between the CD8^+^ T cell response and the duration of the GFD needs to be interpreted cautiously because the GFD compliance may differ among patients. With a good adherence to the GFD, it is expected a decline in antigen-specific memory T cells as the time from the initial immunological response increases. This probably justifies the lack of the T cell gluten-response in the patient who had been following a GFD for 25 years, which is concordant with previous observations [5]. Based on these findings, a negative result could not be considered valid when testing patients who follow a strict GFD for such a long time. Therefore, we excluded that patient to calculate the accuracy of the proposed diagnostic test.

We also investigated the subset of TCRγδ^+^ T cells. They are also mobilized after gluten challenge and do so at a similar magnitude than CD8^+^ T cells. However, TCRγδ^+^ T cells appear at low frequency in blood and the absolute numbers of the CD103^+^ β7^hi^ CD38^+^ TCRγδ^+^ T cells are in some CD patients as low as 2. Moreover, sensitivity and specificity of the proposed CD8^+^ T cell-based test are not increased by including TCRγδ^+^ as a marker. Therefore, this cell subset is not recommended for CD diagnosis, although our work adds evidence of its active role in CD pathogenesis [30, 31]. Remarkably, gut-homing TCRγδ^+^ and CD8^+^ T cells detected in the blood of CD patients after gluten challenge have been described as displaying a T cell repertoire partially overlapping with intraepithelial lymphocytes in the gut [32].

Leonard et al. observed quite similar sensitivity when evaluating the CD8^+^ T cell response and the production of IFN-γ measured by ELISPOT [7]. This is supported by our study, whose results warrant a formal comparison between the two diagnostic tests in HLA-DQ2.5^+^ patients at clinical practice. However, to be implemented into clinical practice, the analysis of gut-homing CD8^+^ T cells by flow cytometry shows numerous advantages over IFN-γ ELISPOT, being the most notorious: much faster processing and result delivery; easier to learn, teach, and use; need of devices (flow cytometer) routinely used in clinical settings; and non-requirement of gliadin peptides, probably allowing accurate diagnosis for subjects with different HLA-DQ receptors.

Besides the clear impact in CD diagnosis and clinical practice of our findings, our work corroborates the critical role of immunological memory in autoimmunity. The longevity and active functionality of memory T cells upon antigen rechallenge perpetuate histological damage and make it a chronic disease. Long-lived memory T cells carry TCRs that recognize antigens more effectively. On antigen re-exposures, they are expanded and mount a highly efficient quicker and stronger immune response. Therefore, these observations must be also considered when investigating new therapeutic targets.

## Conclusions

Activated gut-homing CD8^+^ T cell measurement is a highly accurate blood test for CD diagnosis in patients on a GFD, being of easy implementation in daily clinical practice. Further multicenter studies on a larger patient sample are warranted to confirm the test diagnostic accuracy, mainly in non-HLA-DQ2.5 subjects.

## Supplementary Information

**Additional file 1.** Supplementary materials: Materials S1. Flow cytometry studies in blood.

**Additional file 2.** Figure S1. Gating strategy for detection of the studied T cell populations.

**Additional file 3.** Supplementary materials: Materials S2. IFN-γ ELISPOT (enzyme-linked immunospot) assay. Materials S3. Additional methods: Histological analysis. Intraepithelial lymphogram. Supplementary results: Table S1. Median (IQR) of the studied CD8+ (percentage at day 6 and ratio day 6/day 0) and TCRγδ+ (percentage at day 6) T cell populations in the different groups of participants (in bold) and adjusted p values of the comparisons between groups obtained using a Dunn’s test following a significant Kruskal-Wallis test. Table S2. Global VAS score and VAS of individual clinical symptoms reported by the participants during the six days of the study (mean intensity ± SE and number and percentage of patients reporting the symptom).

**Additional file 4.** Figure S2. Clinical response to gluten challenge.

## Data Availability

The datasets used and/or analyzed during the current study are available from the corresponding author on reasonable request.
