# Supplementary material for: Activated gut-homing CD8+ T cells for coeliac disease diagnosis on a gluten-free diet
Source: BMC Med. 2021 Oct 6;19:237. doi: 10.1186/s12916-021-02116-z (PMC8493675; doi:10.1186/s12916-021-02116-z)
Supplement: Supplementary file 1 — Additional file 1. Supplementary materials: Materials S1. Flow cytometry studies in blood. [file 12916_2021_2116_MOESM1_ESM.docx]

**Additional file 1**

**Activated gut-homing CD8^+^ T cells for coeliac disease diagnosis on a gluten-free diet**

Fernando Fernández-Bañares^1,2*^, Natalia López-Palacios^3*^, María Corzo^4^, Beatriz Arau^1,2^, Mercedes Rubio^4^, Marta Fernández-Prieto^4^, Eva Tristán^1,2^, Mar Pujals^1^, Sergio Farrais^5^, Saúl Horta^4^, Juana María Hernández^1^, Marta Gomez-Perosanz^6^, Pedro A Reche^6^, María Esteve^1,2^ and Concepción Núñez^4^

^*^Fernando Fernández-Bañares and Natalia López-Palacios should be considered joint first authors

^1^Department of Gastroenterology, Hospital Universitari Mutua Terrassa, Terrassa (Barcelona), Spain

^2^Centro de Investigación Biomédica en Red de Enfermedades Hepáticas y Digestivas (CIBERehd), Instituto de Salud Carlos III, Madrid, Spain

^3^Servicio de Aparato Digestivo, Hospital Clínico San Carlos, Instituto de Investigación Sanitaria del Hospital Clínico San Carlos (IdISSC), 28040 Madrid, Spain

^4^Laboratorio de Investigación en Genética de enfermedades complejas, Hospital Clínico San Carlos, Instituto de Investigación Sanitaria del Hospital Clínico San Carlos (IdISSC), 28040 Madrid, Spain

^5^Servicio de Aparato Digestivo, Hospital Universitario Fundación Jiménez Díaz, 28040 Madrid, Spain

^6^Facultad de Medicina, Laboratorio de Inmunomedicina, Departamento de Inmunología, Universidad Complutense de Madrid, 28040 Madrid, Spain

**SUPPLEMENTARY MATERIALS**

**Materials S1. Flow cytometry studies in blood**

Samples were analysed the same day of blood extraction or shipped in the same tube of collection and at room temperature from one participant hospital to the other and analysed the day after sample collection. No differences were observed in the samples analysed in parallel in consecutive days.

Whole peripheral blood (350 µl) was labelled with 4.5 µl of each of the following monoclonal antibodies: FITC anti-human CD103 (clone Ber-ACT8) from Becton Dickinson (BD) Biosciences (San José, California, USA); APC/Cyanine7 anti-human CD3 (clone HIT3a), PE anti-human/mouse integrin β7 (clone FIB504), PerCP/Cy5.5 anti-human CD38 (clone HIT2), BV421 anti-human TCR (clone B1) and APC anti-human CD8 (clone SK1), all from BioLegend (San Diego, CA, USA). After 30 min of incubation at 4 °C in the dark, erythrocytes were lysed with 3 ml of FACs Lysing BD Biosciences reagent.
