# Supplementary figures and images for: Activated gut-homing CD8+ T cells for coeliac disease diagnosis on a gluten-free diet
Source: BMC Med. 2021 Oct 6;19:237. doi: 10.1186/s12916-021-02116-z (PMC8493675; doi:10.1186/s12916-021-02116-z)

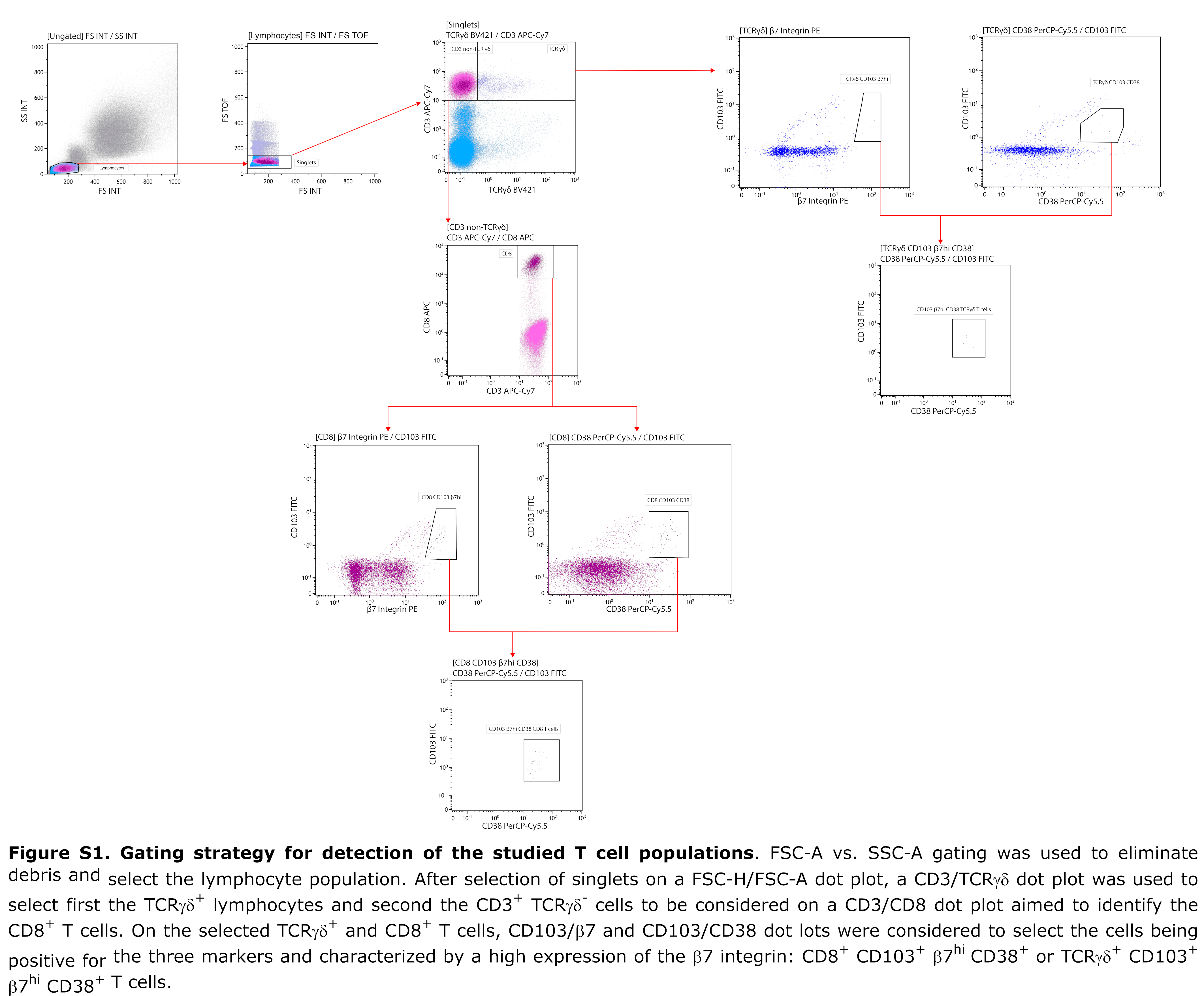

Supplement: Supplementary file 2 — Additional file 2. Figure S1. Gating strategy for detection of the studied T cell populations. [file 12916_2021_2116_MOESM2_ESM.jpg]

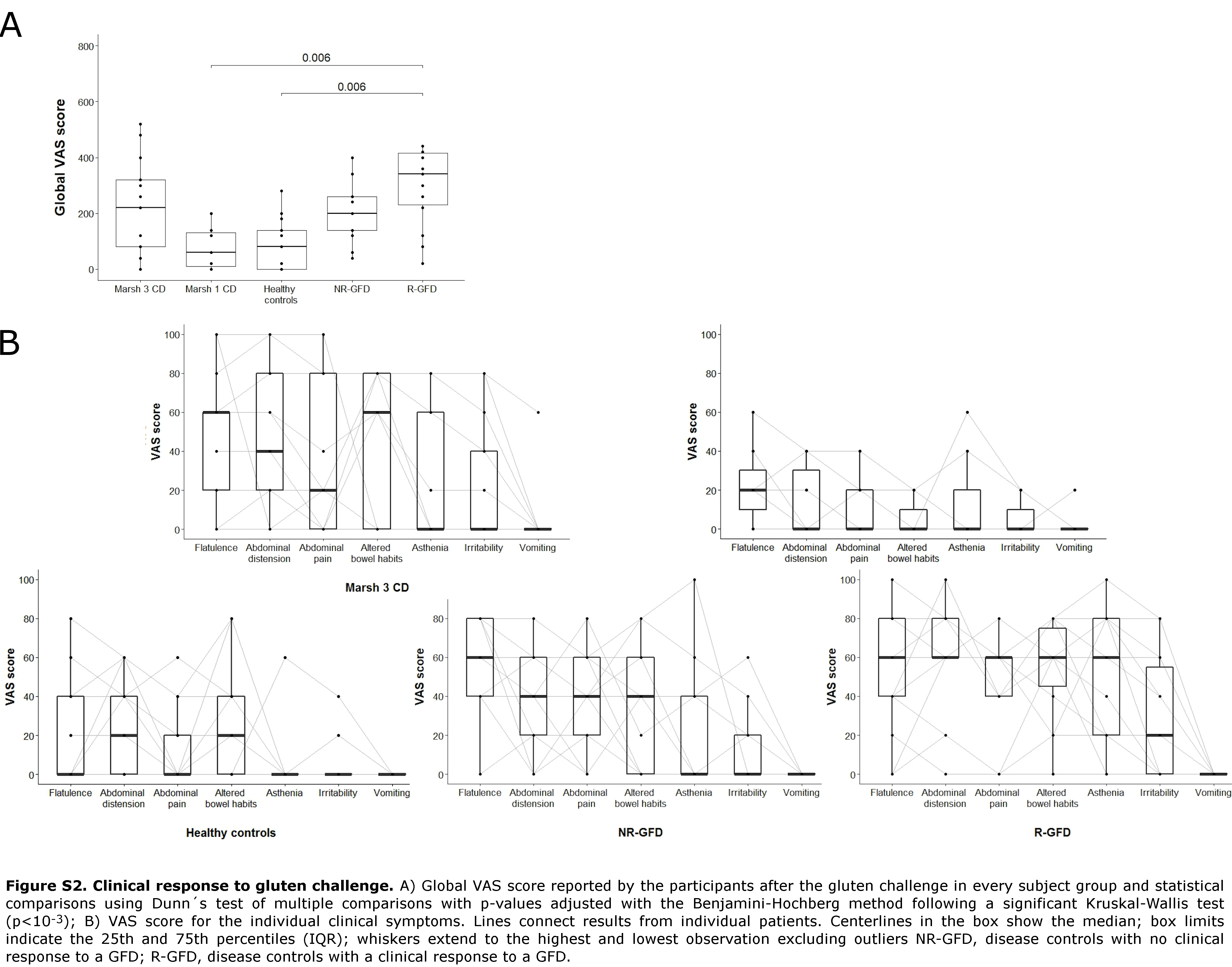

Supplement: Supplementary file 4 — Additional file 4. Figure S2. Clinical response to gluten challenge. [file 12916_2021_2116_MOESM4_ESM.jpg]
