# Supplementary material for: Activated gut-homing CD8+ T cells for coeliac disease diagnosis on a gluten-free diet
Source: BMC Med. 2021 Oct 6;19:237. doi: 10.1186/s12916-021-02116-z (PMC8493675; doi:10.1186/s12916-021-02116-z)
Supplement: Supplementary file 3 — Additional file 3. Supplementary materials: Materials S2. IFN-γ ELISPOT (enzyme-linked immunospot) assay. Materials S3. Additional methods: Histological analysis. Intraepithelial lymphogram. Supplementary results: Table S1. Median (IQR) of the studied CD8+ (percentage at day 6 and ratio day 6/day 0) and TCRγδ+ (percentage at day 6) T cell populations in the different groups of participants (in bold) and adjusted p values of the comparisons between groups obtained using a Dunn’s test following a significant Kruskal-Wallis test. Table S2. Global VAS score and VAS of individual clinical symptoms reported by the participants during the six days of the study (mean intensity ± SE and number and percentage of patients reporting the symptom). [file 12916_2021_2116_MOESM3_ESM.docx]

**Additional file 3**

**Activated gut-homing CD8^+^ T cells for coeliac disease diagnosis on a gluten-free diet**

Fernando Fernández-Bañares^1,2*^, Natalia López-Palacios^3*^, María Corzo^4^, Beatriz Arau^1,2^, Mercedes Rubio^4^, Marta Fernández-Prieto^4^, Eva Tristán^1,2^, Mar Pujals^1^, Sergio Farrais^5^, Saúl Horta^4^, Juana María Hernández^1^, Marta Gomez-Perosanz^6^, Pedro A Reche^6^, María Esteve^1,2^ and Concepción Núñez^4^

^*^Fernando Fernández-Bañares and Natalia López-Palacios should be considered joint first authors

^1^Department of Gastroenterology, Hospital Universitari Mutua Terrassa, Terrassa (Barcelona), Spain

^2^Centro de Investigación Biomédica en Red de Enfermedades Hepáticas y Digestivas (CIBERehd), Instituto de Salud Carlos III, Madrid, Spain

^3^Servicio de Aparato Digestivo, Hospital Clínico San Carlos, Instituto de Investigación Sanitaria del Hospital Clínico San Carlos (IdISSC), 28040 Madrid, Spain

^4^Laboratorio de Investigación en Genética de enfermedades complejas, Hospital Clínico San Carlos, Instituto de Investigación Sanitaria del Hospital Clínico San Carlos (IdISSC), 28040 Madrid, Spain

^5^Servicio de Aparato Digestivo, Hospital Universitario Fundación Jiménez Díaz, 28040 Madrid, Spain

^6^Facultad de Medicina, Laboratorio de Inmunomedicina, Departamento de Inmunología, Universidad Complutense de Madrid, 28040 Madrid, Spain

**SUPPLEMENTARY MATERIALS**

**Materials S2. IFN-γ ELISPOT (enzyme-linked immunospot) assay**

Cryopreserved PBMCs were plated at 200,000 cells/well (50,000 cells/well for positive controls) in ELISpot PVDF plates (MabTech, Nacka Strand, Sweden) that had been previously coated with 15 µg/mL of anti-IFN-γ (MabTech, Nacka Strand, Sweden). Then, PBMCs were stimulated with either 60 μg/mL of the gliadin peptide QLQPFPQPELPYPQPQS (p57-73 QE65) synthetized with purity >90% by Proteogenix (Oberhausbergen, France), 7.5 μg/mL of phytohemagglutin (PHA) (positive control) or left unstimulated (negative control). The gliadin peptide used for stimulation was selected because it includes the most frequent dominant T cell epitopes for HLA-DQ2.5, the HLA class II molecule present in more than 90% of patients with coeliac disease (CD) [18]. All the plates were analysed using the same spot counting procedure and equipment (Cellular Technology Limited Inc., Cleveland, USA) by a researcher blinded to the diagnostic results to obtain the number of IFN-γ secreting cells (SFC, “spot forming cells”). Three replicates were performed for each assay and SFC values were calculated by subtracting means of the negative controls from the mean of the peptide stimulated wells.

**Materials S3. Additional methods**

**Histological analysis**

Two endoscopic biopsies from the bulb and four from the second portion of the duodenum were obtained and placed in separate vials in the index endoscopy for standard histological studies while patients were on a gluten-containing diet. Duodenal samples were processed using haematoxylin/eosin staining. Marsh 1 was considered as an intraepithelial lymphocyte (IEL) count >25 per 100 epithelial nuclei and normal villous architecture.

**Intraepithelial lymphogram**

During the upper gastrointestinal endoscopy, one additional biopsy specimen from duodenum was obtained to analyse IELs by flow cytometry and determine the intraepithelial lymphogram as previously described [15]. In brief, biopsy samples were incubated 90 minutes under agitation with 1 mM EDTA and 1 mM DTT in RPMI 1640 medium supplemented with 10% FCS and antibiotics. After that, the suspension of released cells was collected after centrifugation, washed and stained with the fluorochrome-conjugated antibodies: CD103-FITC (clone Ber-ACT8), TcRγδ-PE (clone 11F2), CD3-PerCP (clone SK7), and CD45-APC (clone HI30), all from Becton Dickinson (BD Biosciences, San Jose, CA, US). The gating strategy selected both TCRγδ^+^ and CD3^-^ cells, which were measured as CD45^+^CD103^+^TCRγδ^+^ and CD45^+^CD103^+^CD3^-^, respectively, over the total CD45^+^CD103^+^ cells. The normal cut-off values for the IEL cytometric pattern in our laboratory are CD3^+^TCRγδ^+^ IEL ≤8.5% (≤mean+2SD) and CD3^-^ IEL ≥10% (10th percentile). These cut-offs were calculated in a sample of 65 non-CD subjects. The intra-assay coefficient of variation was 5.5% (two replicates of each sample processed one immediately after the other), and the inter-sample coefficient of variation was 7.7% (two different samples from each patient obtained in the same procedure). Previous studies have described a high diagnostic accuracy of the intraepithelial lymphogram for CD diagnosis [15, 16, 19, 20].

**SUPPLEMENTARY RESULTS**

**Table S1**. Median (IQR) of the studied CD8^+^ (percentage at day 6 and ratio day 6/day 0) and TCRγδ^+^ (percentage at day 6) T cell populations in the different groups of participants (in bold) and adjusted p values of the comparisons between groups obtained using a Dunn´s test following a significant Kruskal-Wallis test.

|  | **% CD8^+^ CD103^+^ β7^hi^ CD38^+^/total CD8^+^** | **Ratio**  **day 6/day 0** | **% TCRγδ^+^ CD103^+^ β7^hi^ CD38^+^/total TCRγδ^+^** |
| --- | --- | --- | --- |
| Kruskal-Wallis p value | 1.03*10^-07^ | 8.59*10^-05^ | 3.01*10^-06^ |
| **Marsh 3 CD** | **0.27 (0.11-0.51)** | **28.31 (15.43-54.87)** | **0.20 (0.014-0.47)** |
| **vs**. Marsh 1 CD | 0.660 | 0.549 | 0.499 |
| Healthy controls | <10^-3^ | 0.001 | 0.003 |
| NR-GFD | <10^-3^ | 0.001 | <10^-3^ |
| R-GFD | <10^-3^ | 0.004 | <10^-3^ |
| **Marsh 1 CD** | **0.027 (0.015-0.046)** | **6.36 (3.54-7.37)** | **0.031 (0.016-0.060)** |
| **vs.** Healthy controls | 0.003 | 0.019 | 0.053 |
| NR-GFD | 0.002 | 0.028 | 0.010 |
| R-GFD | 0.002 | 0.073 | 0.003 |
| **Healthy controls** | **0.001 (0.0005-0.002)** | **0.39 (0.04-0.85)** | **0.004 (0-0.012)** |
| **vs.** NR-GFD | 1 | 0.711 | 0.559 |
| R-GFD | 0.937 | 0.520 | 0.297 |
| **NR-GFD** | **0.001 (0-0.006)** | **0.63 (0-1.27)** | **0 (0-0.005)** |
| **vs.** R-GFD | 1 | 0.637 | 0.524 |
| **R-GFD** | **0.002 (0-0.003)** | **0.93 (0-1.31)** | **0 (0-0.001)** |

Abbreviations: NR-GFD, disease controls not responding to a gluten-free diet; R-GFD, disease controls responding to a gluten-free diet.

**Table S2**. Global VAS score and VAS of individual clinical symptoms reported by the participants during the six days of the study (mean intensity ± SE and number and percentage of patients reporting the symptom).

|  | **Coeliac disease** | | **Non-CD controls** | | | |
| --- | --- | --- | --- | --- | --- | --- |
|  | **Marsh 3**  **(N=13)** | **Marsh 1**  **(N=7)** | **Healthy (N=13)** | **NR-GFD**  **(N=13)** | **R-GFD**  **(N=14)** |  |
| **Global VAS score** | 223.1 ± 48.1 | 77.1 ± 29.4 | 93.8 ± 25.1 | 195.4 ± 28.9 | 297.1 ± 37.2 |  |
| **No symptoms** | 1 (8%) | 2 (29%) | 4 (31%) | 0 | 0 |  |
| **Flatulence** | 46.2 ± 10  10 (77%) | 22.9 ± 8.1  5 (71%) | 20.0 ± 7.5  6 (46%) | 53.8 ± 6.6  12 (92%) | 57.1 ± 9.3  12 (86%) |  |
| **Abdominal distention** | 46.2 ± 10  11 (85%) | 14.3 ± 7.2  3 (43%) | 26.2 ± 6.9  8 (62%) | 36.9 ± 7.5  10 (77%) | 60.0 ± 7.6  13 (93%) |  |
| **Abdominal pain** | 33.8 ± 10.5  8 (62%) | 11.4 ± 5.9  3 (43%) | 12.3 ± 5.8  4 (31%) | 40.0 ± 6.8  11 (85%) | 45.7 ± 7.1  11 (79%) |  |
| **Altered bowel habits** | 46.2 ± 10.7  8 (62%) | 5.7 ± 3.7  2 (29%) | 24.6 ± 8.2  7 (54%) | 35.4 ± 8.2  9 (69%) | 54.3 ± 6.8  13 (93%) |  |
| **Asthenia** | 24.6 ± 9.9  5 (38%) | 14.3 ± 9.5  2 (29%) | 4.6 ± 4.6  1 (8%) | 18.5 ± 8.9  4 (31%) | 51.4 ± 9.1  12 (86%) |  |
| **Irritability** | 21.5 ± 8.9  5 (38%) | 5.7 ± 3.7  2 (29%) | 6.2 ± 3.5  3 (23%) | 10.8 ± 5.4  4 (31%) | 28.6 ± 8.3  8 (57%) |  |
| **Vomiting** | 4.6 ± 4.6  1 (8%) | 2.9 ± 2.9  1 (14%) | 0  0 | 0  0 | 0  0 |  |

Abbreviations: NR-GFD, disease controls with no clinical response to a GFD; R-GFD, disease controls with a clinical response to a GFD.

Every symptom was scored from 0 to 100 points.

Twelve participants (4 CD patients and 8 NR-GFD disease controls) did not provide these data; instead, they self-reported their symptoms every day during the six days of challenge.
